# *In silico vs in vitro* analysis of primer specificity for the detection of *Gardnerella vaginalis*, *Atopobium vaginae* and *Lactobacillus* spp.

**DOI:** 10.1186/1756-0500-5-637

**Published:** 2012-11-15

**Authors:** Ana Henriques, Tatiana Cereija, António Machado, Nuno Cerca

**Affiliations:** 1 CEB-IBB, Centro de Engenharia Biológica - Instituto de Biotecnologia e Bioengenharia, Universidade do Minho, Campus de Gualtar, 4710-057, Braga, Portugal

**Keywords:** Primer specificity, Primer sensitivity, *In silico* primer testing, *In vitro* primer testing, *G. vaginalis*, *A. vaginae*, *Lactobacillus* spp.

## Abstract

**Background:**

Bacterial vaginosis (BV) is a common pathology of women in reproductive age that can lead to serious health complications, and is associated with shifts in the normal microflora from predominance of *Lactobacillus* spp. to a proliferation of other anaerobes such as *G. vaginalis* and *A vaginae*, which can be detected by PCR. The optimal PCR pathogen detection assay relies mainly on the specificity and sensitivity of the primers used.

**Findings:**

Here we demonstrate that *in silico* analytical testing of primer specificity is not a synonym to *in vitro* analytical specificity by testing a range of published and newly designed primers with both techniques for the detection of BV-associated microorganisms.

**Conclusions:**

By testing primer *in vitro* specificity with a sufficient range of bacterial strains, we were able to design primers with higher specificity and sensitivity. Also by comparing the results obtained for the newly designed primers with other previously published primers, we confirmed that *in silico* analysis is not sufficient to predict *in vitro* specificity. As such care must be taken when choosing the primers for a detection assay.

## Findings

Bacterial vaginosis (BV) is one of the most common vaginal disorders of women in reproductive age and is reported to be linked to increased risks of pre-term labor, HIV infection, postoperative infection and inflammatory pelvic disease [1]. It has been recognized that this pathology is caused by a shift in the microbial ecosystem colonizing the vagina of healthy women; from a *Lactobacillus* spp. dominated microbial population to the proliferation of other anaerobic microorganisms such as *Gardnerella vaginalis*, *Atopobium vaginae*, *Mobiluncus* spp. among others [2-4]. The etiology of BV remains unknown but a recent study reported that microbial biofilms, complex tridimensional structures that are known to be highly resistant to antimicrobial chemotherapy [5], may play a fundamental role in BV [6]. It has been described that *G. vaginalis* may account for 60 to 90% of the BV biofilm mass, while *A. vaginae* may account for 1 to 40% and *Lactobacillus* spp. for 1 to 5% [7]. While trying to confirm these findings, in biofilms from vaginal samples of Portuguese women, using a quantitative PCR approach, we came across some difficulties related with primer specificity, despite the increase in the number of published papers regarding the use of molecular tools, such as PCR, for the detection of BV-associated microorganism [8-11]. In order to assess the usefulness of the primers for the detection of our target microorganisms used we decided to test the analytical specificity (% of non-target organisms detected) and sensitivity (% of target organisms detected) of the primers used. Being a quick and inexpensive method, *in silico* analysis of primer specificity and sensitibity has been widely used. However this technique poses some limitations, already described for other applications, namelly significant differences between the *in silico* prediction for primer specificity/sensitivity and the actual *in vitro* results [8,9].

To our knowledge the analytical comparison of primer *in silico* and *in vitro* specificity/sensitivity for primers designed for the detection of BV-associated microorganisms has not been studied and with the increase in the use of molecular techniques for the study of BV such information becomes relevant.

We began by selecting primers previously described in the literature specific for *Lactobacillus* spp. [12,13], *G. vaginalis*[8,14] and *A. vaginae*[11,15]. The reported primers specificity was mainly determined by *in silico* analysis using sequence alignment such as BLAST [16]. Whenever *in vitro* specificity was reported, often published details of such specificity determinations were scarce. When confirming primer specificity, using a few collection strains, and the same conditions reported in the literature, we found that some *in silico* primer specificity did not correspond to the *in vitro* specificity (data not shown). To address this, we selected the best primers that we could find in the literature and repeated the *in silico* specificity analysis using ProbeMatch [17]. We also designed new primers (using VectorNTI, version 11.0 and sequences available in the GeneBank databases) (Table 1).

**Table 1:** ***In silico *analysis of primer specificity of the primers used in this study**

**Target**	**Probe**		**Sequence (5′ → 3′)**	**No. of target strains detected**^**a**^	**Sensitivity (%)**^**b**^	**No. of non-target strains detected**^**a**^	**Specificity (%)**^**b**^	**Reference**
*G. vaginalis*	Gard154-454	Fw	CTCTTGGAAACGGGTGGTAA	36 (from 40)	90	1 (from 1032184)	100	This study
		Rv	TTGCTCCCAATCAAAAGCGGT	38 (from 40)	95	94 (from 1032184)	99,99	
	GV1 + 3	Fw	TTACTGGTGTATCACTGTAAGG					[15]
		Rv	CCGTCACAGGCTGAACAGT					
	Gv1 + 2	Fw	TCCTGTCTACCAAGGCATCC					[13]
		Rv	CGTGTGATAACCGTCAGGTG					
*A. vaginae*	Atop109-329	Fw	GAGTAACACGTGGGCAACCT	457 (from 467)	97,86	18390 (from 1031757)	98,22	This study	
		Rv	CCGTGTCTCAGTCCCAATCT	449 (from 467)	96,15	1356 (from 1031757)	99,87		
	AtovagRT3	Fw	GGTGAAGCAGTGGAAACACT	134 (from 467)	28,69	0 (from 1031757)	100	[11]	
		Rv	ATTCGCTTCTGCTCGCGCA	109 (from 467)	23,34	2 (from 1031757)	100		
	Atop167-587	Fw	GCGAATATGGGAAAGCTCCG	117 (from 467)	25,05	0 (from 1031757)	100	[10]	
		Rv	TCATGGCCCAGAAGACCGCC	115 (from 467)	24,63	0 (from 1031757)	100		
*Lactobacillus* spp.	AM Lacto	Fw	TGATGCATAGCCGAGTTGAG	7353 (from 12936)	56,84	2513 (from 1019288)	99,75	This study	
		Rv	AGCCGAAACCCTTCTTCACT	5858 (from 12936)	45,28	1027 (from 1019288)	99,9		
	New Lacto	Fw	TGGAAACAGRTGCTAATACCG	11680 (from 12936)	90,29	7069 (from 1019288)	99,31	[16]	
		Rv	GTCCATTGTGGAAGATTCCC	10113 (from 12936)	78,18	3311 (from 1019288)	99,68		
	S21 + A19	Fw	TGCCTAATACATGCAAGTCGA	9754 (from 12936)	75,4	184796 (from 1019288)	81,87	[17]	
		Rv	GTTTGGGCCGTGTCTCAGT	10157 (from 12936)	78,52	72659 (from 1019288)	92,87		

Empty boxes correspond to the primers that could not be analysed because there are no databases available to encompass the intergenic region between 16S and 23S rRNA genes for the *in silico* analysis.

^a^Calculated using ProbeMatch (last accession, May 2012) with the following data set options: Strain – Both; Source – Both; Size – > 1200 bp; Quality – Both.

^a^Calculated using ProbeMatch (last accession, May 2012) with the following data set options: Strain – Both; Source – Both; Size – > 1200 bp; Quality – Both.

^**b**^**Formula**: Specificity = (nts/Tnts)*100 (nts: **number of non-target strains undetected,** Tnts: **total number of non-target strains tested).**

Sensitivity = (ts/Tts)*100 (ts: **number of target strains detected,** Tts: **total number of target strains tested).**

During *in silico* analysis of the selected primers, we found two main problems: (i) the selected published primers for *G. vaginalis* were designed for the 16S rRNA-encoding DNA and 23S rRNA-encoding DNA intergenic region [8,14] and there are no available databases which include this region that could be used for the *in silico* analysis; (ii) some of the primers for *A. vaginae* and *Lactobacillus* spp. showed low sensitivity *in silico*. In order to confirm our *in silico* analysis, *in vitro* testing of primer specificity was performed, initially with 3 strains of each target (*G.vaginalis*, *A. vaginae* and *Lactobacillus* spp., details in Additional file 1: Table S1), using DyNAnzyme PCR Master Mix 2x (Finnenzymes, Thermo Scientific, Finland). The initial amplification conditions comprised 40 cycles with the following temperatures each cycle: 94°C for 30 seconds, 60°C for 30 seconds, and 72°C for 1 minute. Amplified products were analysed in a 1% agarose gel and stained with Midori Green nucleic acid dye (Nippon Genetics Europe GmbH, Germany). Results of the PCR at 60°C showed low sensitivity and specificity for *Lactobacillus* spp. primers, and as such we decided to optimize this detection with the aim of obtaining a greater specificity. The annealing temperature was then adjusted to 62°C, and the number of strains increased to a total of 12 target strains (per group) and up to 34 non-target strains (including *Pseudomonas aeruginosa*, *Staphylococcus epidermidis*, *Streptococcus agalactiae*, *Enterococcus faecalis*, *Escherichia coli* and *Staphylococcus cohnii)* (Table 2). We used different annealing temperatures, in order to observe the effect that this variation would have in primer sensitivity and specificity, which is information that could facilitate the use of these primers in combination (for multiplex PCR assays for example) for quick and effective PCR detection assay.

**Table 2:** ***In vitro *analysis of primer specificity of the primers used in this study**

**Target**	**Probe**	**Annealing temp(°C)**	**No. of target strains detected**^**a**^	**Sensitivity (%)**^**b**^	**No. of non-target strains detected**^**a**^	**Specificity (%)**^**b**^	**Reference**
*G. vaginalis*	Gard154-454	60	3 (from 3)	100.0	0 (from 6)	100.0	This study
		62	12 (from 12)	100.0	0 (from 34)	100.0	
	GV1 + 3	60	3(from 3)	100.0	0 (from 6)	100.0	[15]
		62	10 (from 12)	83.3	0 (from 34)	100.0	
	Gv1 + 2	60	3 (from 3)	100.0	0 (from 6)	100.0	[13]
		62	11 (from 12)	91.7	0 (from 34)	100.0	
*A. vaginae*	Atop109-329	60	3 (from 3)	100.0	5 (from 6)	16.7	This study
		62	12 (from 12)	100.0	15 (from 24)	37.5	
		66	12 (from 12)	100.0	3 (from 34)	91.2	
	AtovagRT3	60	12 (from 12)	100.0	1 (from 24)	95.8	[11]
		62	12 (from 12)	100.0	2 (from 34)	94.1	
	Atop167-587	60	2 (from 3)	66.7	0 (from 6)	100.0	[10]
		62	11 (from 12)	91.6	4 (from 34)	88.3	
*Lactobacillus spp.*	AM Lacto	60	2 (from 12)	16.7	1 (from 24)	95.8	This study
		58	2 (from 12)	16.7	3 (from 34)	91.2	
	New Lacto	60	5 (from 12)	41.7	18 (from 34)	47,1	[16]
		62	1 (from 12)	8.3	8 (from 24)	66.7	
	S21 + A19	60	5 (from 12)	41.7	24 (from 24)	0	[17]
		62	6 (from 12)	50.0	34 (from 34)	0	

^a^Calculated using ProbeMatch (last accession, May 2012) with the following data set options: Strain – Both; Source – Both; Size – > 1200 bp; Quality – Both.

^**b**^**Formula**: Specificity = (nts/Tnts)*100 (nts: **number of non-target strains undetected,** Tnts: **total number of non-target strains tested).**

Sensitivity = (ts/Tts)*100 (ts: **number of target strains detected,** Tts: **total number of target strains tested).**

When comparing *in silico* predictions against *in vitro* results, we found considerable differences in specificity and sensitivity values. Despite the fact that theoretical melting temperature of the *A. vaginae* primers designed in this study was 60°C, at this temperature primer specificity was very low. Only with higher annealing temperature (66°C), we could achieve reasonable specificity (91.2%) and sensitivity (100%) values. The primers for *Lactobacillus* spp. were the ones with the lowest specificity. Of note, some of the primers previously reported as specific were found to be non-specific (0%) despite an *in silico* prediction of 81% of specificity. A lower specificity was expected since these primers were designed for the identification of a genus, which results in a higher inherent genetic variability than primers for the identification a species. However, the accentuated decrease between *in silico* and *in vitro* primer specificity was not anticipated. The primers described by Pepin *et al.* (10) revealed the highest specificity for *A. vaginae* (Table 2), probably due to the previous *in vitro* specificity analysis against at least 20 isolates that was performed by the authors [10]. Although *A. vaginae* primers demonstrated an overall lower specificity, they proved to be highly sensitive, being able to detect all the targets used (Table 2). It is worth to note that the primer designed for this study for the detection of *G. vaginalis* proved to have a higher sensitivity than that of published primers, and that contrary to the results obtained for the other target microorganisms, the *in silico* prediction of sensitivity and specificity was similar to the *in vitro* results obtained.

It is interesting to note that *in silico* analysis for primers specificity was not a good predictor of *in vitro* primer specificity, something already reported by other researchers [8,9], *in silico* predictions do not take into account the chemical reactions/limitations that can occur in the PCR tube and as such cannot truly approximate experimental events. In most articles describing the detection of BV-associated microorganisms the primer specificity testing is described as being performed *in silico*[10-15]. This work confirms the importance of proper *in vitro* analysis of primer specificity, as the *in silico* analysis can sometimes underestimate the possible cross reaction with non-sense strains or species which are not yet sequenced, as is the case of most of the strains present in vaginal environment. Furthermore, the new *G. vaginalis* and *A. vaginae* primers reported here have high *in vitro* specificity, demonstrating its potential for use in clinical microbiology.

## Competing interests

The authors declare that they have no competing interests in this work. The funding organization did not play any role in the design of the experiments and article drafting.

## Authors’ contributions

This an original work that has not been published elsewhere. The experiments were designed by NC and AH, the *in vitro* analysis was done by AH and TC, primer design and *in silico* analysis was done by AM. AH drafted the manuscript, and all authors critically reviewed and agreed with the present manuscript.

## Supplementary Material

Additional file 1 **Table S1.** Description of name and origin of strains used in this study for the *in vitro *testing of primer specificity and sensitivity. Click here for file
