# Supplementary material for: In silico vs in vitro analysis of primer specificity for the detection of Gardnerella vaginalis, Atopobium vaginae and Lactobacillus spp
Source: BMC Res Notes. 2012 Nov 15;5:637. doi: 10.1186/1756-0500-5-637 (PMC3522034; doi:10.1186/1756-0500-5-637)
Supplement: Additional file 1 — Table S1. Description of name and origin of strains used in this study for the in vitro testing of primer specificity and sensitivity. [file 1756-0500-5-637-S1.doc]

**Supplementary material**

**Table S1:** Description of name and origin of strains used in this study for the *in vitro* testing of primer specificity and sensitivity

| **Name of strain** | **Gift from** |
| --- | --- |
| ***Gardnerella vaginalis*** |  |
| AMD | Jefferson, K., Virginia Commonwealth University, USA |
| GV 5-1 | Jefferson, K., Virginia Commonwealth University, USA |
| GV 101 | Jefferson, K., Virginia Commonwealth University, USA |
| VMF1821CO231 | Vaneechoutte M, University of Gent, Belgium |
| VMP1327COL41 | Vaneechoutte M, University of Gent, Belgium |
| VMF1800SVT21B | Vaneechoutte M, University of Gent, Belgium |
| VMF1327COL41 | Vaneechoutte M, University of Gent, Belgium |
| VMF1800SVT31 | Vaneechoutte M, University of Gent, Belgium |
| VMF2213W31 | Vaneechoutte M, University of Gent, Belgium |
| VMF0907COL31 | Vaneechoutte M, University of Gent, Belgium |
| VMF09005VS31 | Vaneechoutte M, University of Gent, Belgium |
| GV UM008 | Cerca, N, DEB-UM |
| ***Atopobium vaginae*** |  |
| BV S065 | Vaneechoutte M, University of Gent, Belgium |
| FB106b | Vaneechoutte M, University of Gent, Belgium |
| VMF914COL43 | Vaneechoutte M, University of Gent, Belgium |
| VMF914COL13 | Vaneechoutte M, University of Gent, Belgium |
| VMF0907COL23 | Vaneechoutte M, University of Gernt, Belgium |
| PB2003/017-T1-2 | Vaneechoutte M, University of Gent, Belgium |
| BV S067 | Vaneechoutte M, University of Gent, Belgium |
| BV S069 | Vaneechoutte M, University of Gent, Belgium |
| FB145-BA-14A | Vaneechoutte M, University of Gent, Belgium |
| FB158-CNA-2C | Vaneechoutte M, University of Gent, Belgium |
| FB130-CNAB-2aD | Vaneechoutte M, University of Gent, Belgium |
| ***Lactobacillus* app.** |  |
| *L. gasseri* UM010 | Cerca, N, DEB-UM |
| *L. pentosus* CECT 4023 | Rodrigues, L, DEB-UM |
| *L. casei* CECT 5275 | Rodrigues, L, DEB-UM |
| *L. rhamnosus* CECT 288 | Rodrigues, L, DEB-UM |
| *L. coryniformis subsp. torquens* CECT 4129 | Rodrigues, L, DEB-UM |
| L. paracasei 227 | Rodrigues, L, DEB-UM |
| *L. agilis* CCUG 31450 | University of Gent, Belgium |
| *L. animalis* ATTC 35046 | University of Gent, Belgium |
| *L. bifermentans* ATTC 35409 | University of Gent, Belgium |
| L. brevis ATTC 14869 | University of Gent, Belgium |
| L. bruchneri ATTC 4005 | University of Gent, Belgium |
| L. fructivorans ATTC 8288 | University of Gent, Belgium |
| **Other strains** |  |
| *Staphylococcus* spp. UM | Cerca, N, DEB-UM |
| *Streptococcus* spp. UM | Cerca, N, DEB-UM |
| *Klebsiella* spp. UM | Cerca, N, DEB-UM |
| *Gemella* spp. UM | Cerca, N, DEB-UM |
| *Bacillus* spp.UM | Cerca, N, DEB-UM |
| *S. agalactiae* UM | Cerca, N, DEB-UM |
| *Enterococcus faecalis* UM016 | Cerca, N, DEB-UM |
| *Pseudomonas aeruginosa* | DEB-UM |
| *Escherichia coli* | DEB-UM |
| *Staphylococcus epidermidis* 9142 | Cerca, N, DEB-UM |
